# Sanitas Oil

**Published:** 1886-06

**Authors:** E. S. Talbot


### SANITAS OIL.

BY E. S. TALBOT, M. D., D. D. S.

In the April number of the INDEPENDENT PRACTITIONER Dr. Harlan quotes from the opinions of several eminent gentlemen to substantiate his statement that Sanitas Oil is a disinfectant, and reports experiments made by Prof. Chas. B. Gibson, of the College of Physicians and Surgeons. With all respect for Dr. Gibson's acknowledged ability as a chemist, I found that the result of his experiments only confirmed my opinion, as any disinfectant which requires from twenty-four to thirty-six hours to make an impression on a substance rates so low in the list of disinfectants that its good-for-nothingness would make it useless. I think we are all agreed "that a disinfectant is capable of combining with the gases and other products of decomposition, and so removing it." I have made experiments with Sanitas, both sealed and unsealed, with and without a reagent. Some of them have covered from two to three months time. I procured the Sanitas from the importers, Sargent

& Co., and the S. S. White Dental Manufacturing Co., Chicago, and both informed me that this was the genuine article of commerce, manufactured in England. I admit it to be essential to seal the bottles or cavities, as the $H_2$ S would otherwise escape free. Contradicting Dr. Harlan's definition of a disinfectant, even when sealed, the odor of $H_2$ S is very apparent upon unsealing, the Sanitas having acted so slowly, if at all, that the sulphuretted hydrogen responds to the reagent. In experiments where a reagent (acetate of lead) was used, apparently no chemical change took place, further proving that Sanitas Oil had not "entered or united chemically with $H_2$ S," consequently was not a disinfectant. I procured two specimens of Sanitas, one from E. H. Sargent & Co., and one from S. S. White's Dental Manufacturing Co., which I sent to Prof. Haines for examination as to their disinfecting powers, and I present below his reply:

CHICAGO, May 5, 1886.

EUGENE S. TALBOT, M. D., D. D. S.

*Dear Sir:*—The two specimens of Sanitas Oil placed by you in my hands a few weeks since, have been subjected to tests to determine their disinfecting powers, with results as given below:

A solution in distilled water of sulphuretted hydrogen ($H_2$ S) was prepared of such strength that each volume of the liquid held in solution exactly three volumes of the gas. Four wide-mouth bottles, each of one ounce capacity, were procured, and ten cubic centimeters of the sulphuretted hydrogen solution were placed in each. To the first of these bottles (labeled No. 1), five cubic centimeters of the Sanitas Oil (bearing E. H. Sargent & Co.'s label) were added; to the second (No. 2), a like quantity of ordinary oil of turpentine; to the third (No. 3), the same amount of hydrant water; and to the fourth (No. 4), the same quantity of the officinal liquor soda chlorate (U. S. P.). The sulphuretted hydrogen was at once wholly destroyed in No. 4, no trace of it being discoverable by any test, thus illustrating the action upon $H_2$ S of a well recognized and thoroughly active disinfectant. In the other three bottles, however, the sulphuretted hydrogen remained strongly marked. They were accordingly stopped with tightly fitting corks, and were vigorously shaken every two or three hours during the working part of the day, the corks being removed for a few seconds both immediately before and after the agitation, so as to allow the free entrance of air. Twice each day, morning and afternoon, sulphuretted hydrogen was tested for in each bottle, by moistening a strip of filtering paper with a ten per cent. solution of acetate of lead and suspending it above the liquid for half a minute. When no darkening of the acetate was observed upon applying this test, the sulphuretted hydrogen was said to be destroyed, and the time was noted. At the end of three and one-half days the sulphuretted hydrogen had disappeared from No. 2 (to which oil of turpentine had been added); at the end of seven and one-half days $H_2 S$ was no longer present in No. 3 (to which hydrant water had been added); while the destruction of the sulphuretted hydrogen was not complete in No. 1 (the one containing Sanitas Oil) until nine days had passed by.

An exactly similiar series of experiments was performed with the other specimen of Sanitas Oil you sent me (the one bearing the label of The S. S. White Dental Co.), except that *ten* cubic centimeters of Sanitas Oil, of oil of turpentine, of hydrant water and of chlorinated soda solution were used instead of the *five* cubic centimeters of these substances that were employed before. The results were quite similar to those obtained in the first experiments. In No. 4 (to which the chlorinated soda was added) the sulphuretted hydrogen was destroyed at once; in No. 2 (containing oil of turpentine) it had disappeared at the end of four days; in No. 3 (to which hydrant water had been added) it was gone in seven days; and it was not destroyed in No. 1 (containing Sanitas Oil) until the end of the ninth day.

The conclusion to be drawn from the above experiments is obvious. Sanitas Oil (at least the two specimens of it I received from you) is possessed of exceedingly feeble disinfecting powers, being almost infinitely less active than solution of chlorinated soda, being less energetic than oil of turpentine, and is surpassed in its activity even by ordinary water.

Yours respectfully,

WALTER S. HAINES, M. D.,

Prof. of Chemistry in Rush Medical College.

The experiments made by Prof. Haines corroborate mine. They have all been conducted with the Sanitas of commerce in this country. We have no means of knowing the variety of Sanitas experimented with by the gentlemen abroad, mentioned by Dr. Harlan.

